# Bibliography

**Published:** 1896-10

**Authors:** 


### Bibliography.

Transactions of the American Dental Association at the thirty-fifth annual session, held at Asbury Park, N. J., 1895. S. S. White Dental Manufacturing Company, 1886.

This report, while very much delayed, contains an unusual amount of valuable matter. The illustrations are very superior and are a credit to the publisher. There has been much criticism over the delay of issuing this volume, and, while this is a matter of regret, very few, it is imagined, realize the amount of work connected with the preparation of a volume of 380 pages. It would be poor policy to adopt careless methods, which speed is sure to engender, in place of that careful preparation manifested in this report.

Extraction of the Teeth. By J. F. Colyer, L.R.C.P., M.R.C.S., L.D.S., Dental Surgeon and Lecturer on Dental Surgery to Charing Cross Hospital, etc. London: Claudius Ash & Sons, Limited.

The author of this small book of 102 pages, fully illustrated, says, "I have published the following notes on 'Extraction of the

Teeth' in the hope that they may prove of some small value to the student and practitioner."

To the beginner in practice the book will certainly prove an invaluable companion. It is prepared with care, and the necessary manipulations are clearly described, so that no one should experience difficulty in practically following them, and what is more to the purpose, they are correctly stated.

It will probably never be possible to lay the forceps entirely aside in dental practice, and a dental manual of this kind not only has a special value, but is an absolute necessity.

Chapter III., on the "Extraction of Misplaced Teeth," might well have been omitted, and should not have a place in future editions. The extraction of the central incisor because it may be outside of the arch or the lateral inside is contrary to American thought and practice. The same may be said of the cuspidate.

Chapter V., on "Difficulties, Complications, and Sequelæ of Extraction of the Teeth," must prove of special value to the beginner.

As a whole, the book can be cordially recommended for reference in this special line of work.

